# Comparative analysis of sitting pressures in individuals with spinal cord injury: static vs. dynamic air cushions

**DOI:** 10.3389/fpubh.2025.1637106

**Published:** 2025-12-15

**Authors:** Irene Corral-López, Adrià Marco-Ahulló, César Rubio-Belmonte, Xurxo Segura-Navarro, Juan Manuel Pozo-Alegre, Lluïsa Montesinos-Magraner

**Affiliations:** 1 Spinal Cord Injury Unit, Physical Medicine and Rehabilitation Service, Vall d’Hebron Barcelona Hospital Campus, Barcelona, Spain; 2 Department of Personality, Treatments and Methodology, Faculty of Psychology, Universidad Católica de Valencia “San Vicente Mártir”, Valencia, Spain; 3 University and Polytechnic La Fe Hospital, Valencia, Spain

**Keywords:** spinal cord injury, pressure injuries, anti-decubitus cushion, Nubolo®|Med, prevention

## Abstract

**Background:**

Pressure injuries (PIs) significantly affect the health and social participation of individuals with spinal cord injury (SCI). Anti-decubitus cushions help prevent these injuries by distributing weight and reducing peak pressures. Evidence supports air cushions as the most effective solution for pressure relief during sitting. In recent years, in order to enhance their effectiveness, these cushions have incorporated dynamic and intelligent systems.

**Objective:**

To analyse and compare sitting pressure values while using a commercially available dynamic intelligent air cushion—specifically the Nubolo®|Med—versus other high-end air cushions. Methods: The sample comprised 41 individuals with chronic, complete traumatic SCI, presenting various risk factors for pressure injuries. Measurements were conducted using the Nubolo®|Med cushion, participants’ usual high-end air cushions, and a CONFORMatSYSTEM mat for pressure recording. Outcome variables were peak pressure (PP), normalized peak pressure (PP/CA), average pressure (AP), and contact area (CA), measured on the Nubolo®|Med and on participants’ usual cushion. A comparative analysis was subsequently carried out between the two conditions.

**Results:**

No significant differences were found between the two conditions in the AP and CA variables. However, statistically significant differences were observed in the PP [static cushion: 38.04(12.46) mmHg vs. Nubolo®|Med: 31.81(9.57) mmHg; *z* = 4.38, *p* < 0.001, *r* = −0.73] and PP/CA [static cushion: 0.080(0.024) mmHg/mm^2^ vs. Nubolo®|Med: 0.065(0.016) mmHg/mm^2^; *z* = −3.99, *p* < 0.001, *r* = 0.67] variables.

**Conclusion:**

The findings indicate that the Nubolo®|Med cushion showed lower peak sitting pressures compared to high-end static air cushions in individuals with complete SCI.

## 1 Introduction

Sustaining a spinal cord injury (SCI) entails the risk of developing secondary complications such as musculoskeletal or cardiorespiratory issues, autonomic dysreflexia, or pain, among others (1, 2). Pressure injuries (PIs) are one of the main secondary consequences of SCI, occurring in the acute phase of the injury as well as during active rehabilitation and in the long term (3, 4). PIs are generally defined as damage to the skin area due to prolonged exposure to pressure exceeding capillary pressure, causing a localised area of ischaemic necrosis of the soft tissues (5, 6). It should also be noted that PIs may not only result from prolonged exposure to pressure but can also be caused by brief episodes of elevated peak pressure (6). In addition, other extrinsic factors such as shear forces, friction, moisture, and temperature, together with intrinsic factors including nutritional status, comorbidities, and impaired mobility, contribute to the onset and progression of these injuries (7).

In economic terms, the treatment and management of these injuries place a significant burden on the healthcare system (8). In this regard, Malekzadeh et al. (9) highlighted in their meta-analysis that PIs are one of the most significant determinants of the cost of care in individuals with SCI. Complementing this evidence, Wijker et al. (10) recently analyzed electronic health records from a Dutch rehabilitation center focusing on patients with SCI, and reported a mean annual rehabilitation cost of €6,368 (USD $6,956) per patient, with a mean total cost per patient of €15,412 (USD $16,836) across the follow-up period. These findings underscore the considerable economic impact of PIs in this population.

Additionally, the negative impact on patients’ social, occupational, and quality of life domains is considerable, as treatment requires prolonged offloading of the affected area and, therefore, complete bed rest (11). This situation may significantly affect the level of social participation of those who develop such injuries, potentially increasing existing morbidity and leading to mental health issues (4, 12).

Individuals with SCI appear particularly vulnerable to developing such injuries due to motor and sensory deficits and/or changes in body composition (13, 14). In fact, a recent meta-analysis observed that one in three people with SCI develop PIs (15). Various studies have identified the risk factors associated with PIs in people with mobility impairments, particularly those with SCI. These works highlight factors such as skin quality and care, mobility, characteristics of the SCI, tobacco use, nutritional status, previous history of PIs, and the patient’s level of independence in managing the condition (16, 17). Nevertheless, numerous studies and clinical guidelines on PI prevention and management concur that daily monitoring of skin status and regular repositioning in both the wheelchair and bed (along with the material properties of support surfaces) are essential in preventing such injuries (18–20).

### 1.1 Related works

As previously mentioned, the seating surface is one of the factors influencing the occurrence of PIs. In the case of individuals with complete SCI, particularly at cervical and thoracic levels, this is a determining factor as they spend most of the day seated in a wheelchair. For this reason, several scientific studies have focused on assessing the pressure-relieving effectiveness of different types of cushions made from various materials by analysing peak pressure values (21–23).

He and Shi (22) conducted a rapid review and showed that static air cushions outperform gel and foam cushions in reducing interface pressures. Nevertheless, recent technological advances have also impacted on the field of anti-decubitus cushions. In this context, new models of intelligent and dynamic cushions are emerging, necessitating assessment of their effectiveness in pressure reduction compared to high-end static air cushions, which are currently the product of choice for patients with the highest vulnerability to PI development.

Moving towards these active solutions, Fadil et al. (24) designed and evaluated a prototype dynamic cushion composed of multiple air cells that inflated and deflated alternately. Their findings demonstrated promising results in redistributing interface pressures over time, suggesting potential benefits for the prevention of PI. Similarly, Arias-Guzman et al. (25) examined a smart redistribution cushion in wheelchair users with spinal cord injury. This device was capable of automatically adjusting pressure according to risk areas detected, and the study reported significant improvements in gluteal tissue oxygenation compared with conventional pressure relief maneuvers. However, both studies focused on devices under development or experimental settings, rather than evaluating commercially available dynamic cushions. In fact, a recent comprehensive review by Mansouri et al. (23) concluded that although numerous dynamic and intelligent prototypes have been proposed in the literature, there is a critical lack of clinical evidence involving commercial dynamic cushions.

Based on the aforementioned considerations, the main objective of this study was to analyse and compare variables related to seated pressures (peak pressure in the contact area [PP], normalized peak pressure [PP/CA], average pressure [AP], and contact area [CA]) using a dynamic intelligent commercial air cushion (Nubolo®|Med), in comparison with a high-end static air cushion.

## 2 Materials and methods

### 2.1 Design

This was a cross-sectional descriptive study in which pressure data were compared from the same individuals with chronic SCI at two different time points: (i) while using the Nubolo®|Med cushion and (ii) while seated on their usual air cushion (always classified as high-end).

### 2.2 Participants

The total sample included 41 participants. Participant recruitment was non-probabilistic and by convenience. Sample size was calculated *a priori* using G*Power 3.1 software (University of Düsseldorf, Düsseldorf, Germany), with a Cohen’s d effect size of 0.45 [based on previously published data (18)]. The significance level was set at 0.05 and statistical power at 0.8.

Participant eligibility—voluntary, anonymous, and uncompensated—was subject to the following inclusion criteria: (i) complete SCI at cervical (tetraplegia) or thoracic (paraplegia) level for at least 1 year; (ii) habitual use of a high-end air cushion as an assistive device for PI prevention; (iii) previous history of PIs or high risk of developing them (e.g., due to structural deformity or pelvic obliquity); and (iv) aged between 18 and 65 years.

To describe the sample in greater depth: gender distribution was 33/8 (with higher male prevalence, 80.5%), mean age was 46.17 (11.06) years, mean weight was 74.34 (14.09) kg, and 78.05% had a thoracic-level lesion, with the remainder at the cervical level. Regarding clinical history related to PIs, 73.17% showed alterations in at least one bony structure in the seated area (e.g., irregular edges, bone spicules, bone steps…), 82.92% had a history of PIs, and 46.34% had undergone surgery for this reason. Additionally, Table 1 presents the characteristics of the participants, stratified by injury level (cervical or thoracic).

**Table 1:** Participant characteristics by level of SCI.

Level of spinal cord injury	Gender (male/female)	Age (years)	Weight (Kg)	History of PI (yes/no)	Bone structure alterations (yes/no)	Previous surgery (yes/no)	Wheelchair (manual/powered)
Thoracic (*n* = 32)	26/6	45, 34 (10, 88)	74, 45 (14, 93)	27/5	25/7	15/17	28/4
Cervical (*n* = 9)	7/2	49, 11 (11, 85)	73, 94 (11, 38)	7/2	5/4	4/5	5/4

All participants signed informed consent prior to participation. The study was approved by the Ethics Committee of Hospital Universitari Vall d’Hebron before its commencement [PR(ATR)364/2019].

### 2.3 Instruments

The following were used: Nubolo®|Med cushion (assistive product under evaluation), participants’ prescribed high-end static air cushions, and a CONFORMat SYSTEM pressure mat (pressure measurement device).

The Nubolo®|Med is a dynamic pressure-management device designed to prevent pressure-injuries and provide optimized postural support through continuous load redistribution across the seating interface. The cushion is divided into multiple zones, each containing independently controlled pneumatic chambers actuated by the control unit. Furthermore, the device applies biomechanical interface-pressure modeling to determine initial pressure values, derived from previous pressure-mapping studies. Operationally, minimum effective support pressure is computed using the relationship *P*_min effective_ = $\frac{F}{A}$ , where *F* is user body weight and *A* is the effective contact area, thereby ensuring a baseline pressure support that reduces tissue-collapse risk.

Pressure-mapping analyses identify the ischial region as the area of highest load concentration. Accordingly, the system applies selective pressure unloading in this zone, while compensatory pressure is redistributed to peripheral regions to maintain overall stability. Specifically, the force to be compensated is calculated as *F* = *A*_reduced P zones_ × *P*_reduction_.

From an operational standpoint, dynamic behavior is delivered through alternating inflation/deflation cycles that reach programmed *P*_max_ and *P*_min_ values with defined dwell times. To this end, airflow is managed via solenoid valves, and a compressor provides the required pressure supply. Integrated sensors continuously monitor chamber pressure and detect user postural shifts. To promote tissue perfusion, and to achieve the intended biomechanical massage effect, the system ensures a minimum pressure gradient of *P*_max_ − *P*_min_ ≥ 20 mbar.

The CONFORMat SYSTEM pressure mat, which measures pressure distribution between the human body and support surfaces (e.g., seats, cushions), together with the Tekscan CONFORMat SYSTEM software, allowed for the generation of pressure maps and recording of the variables under study [peak pressure (PP), normalized peak pressure (PP/CA), average pressure (AP), and contact area (CA)], which were subsequently subjected to comparative analysis. The mat was calibrated for each participant following the manufacturer’s guidelines.

### 2.4 Procedure

Participants attended the research centre where the study was conducted and were reminded of the protocol. Following an explanation of the study’s objectives and nature, and the signing of informed consent, the evaluation session began with a short interview recording the following sociodemographic and anthropometric data: (i) age, (ii) weight, (iii) diagnosis and injury level, (iv) presence of sensory impairment, (v) incontinence, (vi) PI history, (vii) previous surgery in the gluteal and/or ischial region, and (viii) usual cushion model.

**Figure 1:**
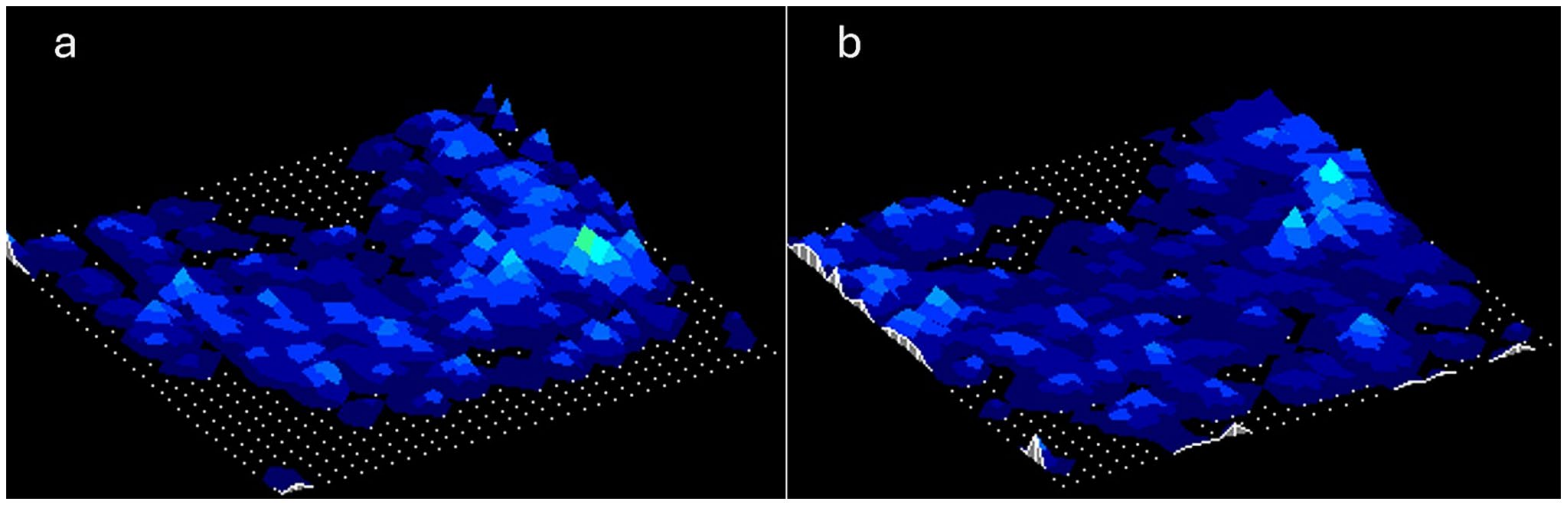
Example of pressure test performed with the high-end static air cell cushion **(a)** and the NUBOLO-MED cushion **(b)**.

In all cases, an ultrasound scan of the seated area (i.e., gluteal and ischial region) was performed to identify bone abnormalities. Pressure measurements were then taken on both seating surfaces: (i) static air cushion and (ii) Nubolo®|Med cushion. All assessments were conducted by trained professionals following a standardised protocol. Two pressure maps were recorded for each subject (Figure 1).

Figure 1 illustrates representative pressure distribution maps obtained from the same participant using both seating surfaces. The visual comparison clearly demonstrates the pressure redistribution differences between the two cushion types. In the high-end static air cushion (Figure 1a), the pressure map shows concentrated high-pressure zones (indicated by lighter/warmer colors) clustered primarily around the ischial tuberosities, with distinct pressure peaks reaching higher intensity values. These concentrated pressure areas represent potential risk zones for pressure injury development due to sustained loading on vulnerable anatomical structures. Conversely, the Nubolo®|Med dynamic cushion (Figure 1b) exhibits a more dispersed pressure distribution pattern, with reduced intensity in peak pressure areas and less pronounced clustering of high-pressure zones. The dynamic redistribution achieved by the intelligent air cell system results in a more uniform pressure spread across the contact surface, effectively reducing localized pressure concentrations that could lead to tissue ischemia.

To measure pressure, the CONFORMat pressure mat was first placed over the participant’s usual static air cushion, followed by placement on the Nubolo®|Med cushion, using the same procedures. The Nubolo®|Med was configured by the technical team according to each participant’s needs (following standard commercial configuration procedures). During measurement, participants were instructed to remain seated with upper limbs in their usual resting position and feet on the footrests for 30 s, the standard duration for pressure registration.

### 2.5 Data analysis

The CONFORMat SYSTEM pressure mat recorded 200 frames in each measurement, during an interval of 30 s (6.67 Hz). Although optimal measurement duration in seating pressure mapping remains undefined in the literature (26), recent studies have used 20–30 s measurement windows in SCI users for pressure mapping assessments (27) demonstrating that short-duration high-frequency acquisition can capture meaningful pressure variations.

Subsequently, the average of each measurement was calculated using the function included in the Tekscan CONFORMat SYSTEM software analysis tool. In order to correctly analyse the records, the seating area of each measurement was selected to exclude any possible pressures derived from the contact between the back and the pressure mat. Once the average was obtained, four variables were calculated: (i) maximum pressure or peak pressure of the contact area (PP), (ii) average pressure of the contact area (AP), measured in mmHg, (iii) contact area (CA), measured in mm^2^, and (iv) normalized peak pressure (PP/CA), calculated as PP divided by CA and expressed in mmHg/mm^2^.

### 2.6 Statistical analysis

A preliminary outlier analysis was conducted on all variables to ensure the robustness of our statistical comparisons. *Z*-scores were calculated for each subject’s, and any case with ∣*z*∣ > 3 was flagged as an outlier. The two-iteration outlier removal procedure was then compared with the IQR method, yielding 100% agreement. Five subjects met this criterion and were excluded from all subsequent analyses. The final sample for comparative testing thus comprised 36 participants, minimizing the influence of extreme values on our results.

Following outlier removal, the Shapiro–Wilk test was applied to each variable to check the assumption of normality. In this regard, a non-parametric distribution of the data was found for all variables. Therefore, the Wilcoxon signed-rank test for related samples was used for pairwise comparison of all the variables studied. The significance level was set at 0.05 for all cases.

Statistical analyses were performed using the Statistical Package for the Social Sciences (SPSS) software, Version 24 (IBM, Chicago, IL, USA).

## 3 Results

After conducting the relevant statistical analyses, no statistically significant differences were found in the AP variable (*z* = 0.24, *p* = 0.81, *r* = 0.04) between the “high-end static cushion” condition [18.77 (4.24) mmHg] and the Nubolo®|Med condition [18.49 (3.31) mmHg]. Similarly, no differences were found in the CA variable (*z* = 0.88, *p* = 0.38, *r* = 0.15) between the “high-end static cushion” condition [505.56 (195.68) mm^2^] and the Nubolo®|Med condition [519.01 (200.00) mm^2^]. However, statistically significant differences were observed in the PP variable between the two conditions (*z* = 4.38, *p* < 0.001, *r* = −0.73), with mean values of 38.04 (12.46) mmHg in the “high-end static cushion” condition and 31.81 (9.57) mmHg for the Nubolo®|Med condition. Additionally, when normalized by CA, PP was significantly lower for Nubolo®|Med [0.065 (0.016) mmHg/mm^2^] compared to the “high-end static cushion” [0.080 (0.024) mmHg/mm^2^] (*z* = −3.99, *p* < 0.001, *r* = 0.67).

**Figure 2:**
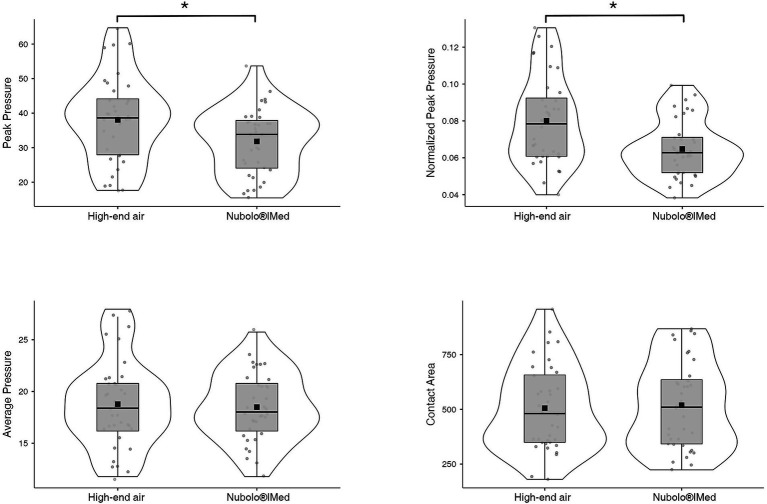
Box plots of peak pressure (PP), normalized peak pressure (PP/CA), average pressure (AP) and contact area (CA) values of static cushions and NUBOLO. *Indicates differences between the types of cushions.

Following methodological standards highlighted in biomedical fields, where the efficacy of novel approaches is systematically supported by comparative tables and figures (28), Figure 2 provide comparative evidence to substantiate the effectiveness of the proposed dynamic cushion. Furthermore, an additional table has been included as a supplementary material, where the differences between the two types of cushions in the studied variables are presented in detail.

## 4 Discussion

As previously mentioned, the main objective of this research was to analyse and compare the variables related to pressures in sitting position [(PP), (AP), and (CA)] when using high-end static air cushions, compared to these pressure values when using the dynamic intelligent air cushion Nubolo®|Med. In this sense, the results of this study showed that there were no statistically significant differences in AP and CA values when comparing these variables between the two conditions. However, statistically significant differences were found in the PP and PP/CA variables, with lower mean values when using the dynamic air cushion Nubolo®|Med.

The findings related to AP and CA are consistent, since if there is no variation in the contact area, there should, in theory, be no differences in AP, as the weight of the subject being evaluated has not changed. On the other hand, observing lower values in PP suggests that the Nubolo®|Med cushion may be more effective in distributing pressure across the contact area.

It is currently considered that pressure and shear forces are the main cause of PI with the duration of time that these forces are applied to tissues being crucial for the physiological changes leading to tissue ischaemia and oedema due to capillary and lymphatic occlusion, ultimately progressing to tissue necrosis. Several studies consider the relationship between the amount of pressure and time as a parabolic curve; that is, skin damage occurs with both low pressures sustained over long periods and high pressures sustained over short periods (29). It is believed that a pressure approximately double the capillary closing pressure, applied for 2 h, causes irreversible ischaemic damage to tissue, and pressures lower than this threshold or of shorter duration are unlikely to cause necrosis (11).

Thus, the desired feature for a cushion designed to reduce pressure in support areas, for the prevention and management of PIs, is its ability not only to reduce the magnitude of localized pressure forces in more vulnerable areas, but also to reduce the time during which such pressures are maintained in those anatomical locations more exposed to injury. This is the value of dynamic cushions, as they can distribute pressures across support areas.

Regarding pressure, the maximum capillary occlusion pressure is considered to be 20 mmHg for practical purposes (although the hydrostatic pressure of venous capillaries is 16 mmHg, while that of arterial capillaries ranges between 33 and 35 mmHg) (30, 31). Thus, pressures above 20 mmHg in a limited area for an extended time can initiate a process of tissue ischaemia that impairs the delivery of oxygen and nutrients to the affected area, leading to tissue degeneration, and if sustained, tissue necrosis (11). However, there is no consensus on the pressures that may precipitate the development of PI. Some studies suggest that pressures above 60–70 mmHg significantly increase the risk of PIs (32), while others suggest the ideal situation is to keep pressures below 35 mmHg in immobile patients and below 60 mmHg in patients who can relieve pressure by elevating or laterally tilting (33). The results of this study show that both types of air cushions studied, high-end static and Nubolo®|Med, maintain an average pressure below 20 mmHg (18.77 mmHg and 18.49 mmHg, respectively), and therefore below the maximum capillary occlusion pressure. Moreover, the PP value obtained for Nubolo®|Med (33.12 mmHg) is slightly below the hydrostatic pressure of arterial capillaries and the optimal value (35 mmHg) for immobile patients, as established in the study by Dover et al. (33), which is not the case for high-end static air cushions, where the mean PP reaches 38.04 mmHg. However, it should be noted that in both conditions, these PP values are below the 60–70 mmHg range, where the risk seems to increase significantly (32), and below the optimal value (60 mmHg) for patients able to relieve pressure by elevating or laterally tilting (33).

Regarding the Time Variable, it is accepted that the origin of PI may, result from both low pressures sustained over prolonged periods and higher pressures maintained for a duration equivalent to or greater than 2 h (29). In this context, seating in individuals with spinal cord injuries and an active lifestyle often extends well beyond this time. The results of this study show that the mean of the maximum/peak pressures (PP = 31.81 mmHg) approaches the hydrostatic pressure of the arterial capillary. However, the dynamic system of the Nubolo®|Med cushion, compared to static air cushions, reduces the duration for which such peak pressures are maintained at a single point, especially at the most vulnerable regions of each user. This effect is achieved through its personalised configuration, which varies both the magnitude and the duration of pressure across the different air cells that compose the cushion. The effectiveness of the Nubolo®|Med dynamic cushion may be partly explained by its capacity to introduce intermittent variations in interface pressure at the ischial tuberosities. Such variations could facilitate periods of pressure relief that may allow microvascular blood flow to be restored, at least transiently, thereby reducing the risk associated with sustained ischemia and hypoxia in the underlying tissues. This interpretation aligns with the aforementioned time–pressure relationship, whereby prolonged loading above the capillary closing pressure increases the likelihood of tissue damage.

Finally, it is worth mentioning some ongoing research in the area of assistive products for individuals at risk of developing PIs, specifically in dynamic and/or intelligent air cushions. In recent times, some research teams have focused on the development and evaluation of these intelligent or dynamic cushions (24, 25). However, it is important to highlight that in these studies, the researchers primarily focused on monitoring the functionality of the device itself and how it was able to perform these changes, comparing it to situations where the subject had to perform postural changes, without comparing it to high-end static air cushions available on the market, which are used by the vast majority of patients with sensory-motor neurological injuries for PI prevention, as has been done in this study.

This study is not without some limitations, which are outlined below, along with suggestions for future research in this area. Firstly, it is worth noting that the sample used for this study is not balanced in terms of sex, as it is predominantly male, which may introduce some bias into the results. However, this distribution corresponds to the prevalence rates of spinal cord injuries between men and women. On the other hand, the participants in the studied sample are at high risk of developing PIs, or, in most cases, have already been treated or surgically intervened for this condition (as detailed in the materials and methods section). This situation means that participants already have an adapted and individualised configuration in their usual static air cushion, implying that the baseline PP recorded is not as high, and therefore the range of improvement is lower than expected if they had not started from this adapted configuration. However, despite the above, it seems that the versatility in the configuration of the Nubolo®|Med improves the results related to PP. Future studies could address the aim of this study at other stages of SCI, during the sitting phase and the active rehabilitation phase, particularly to prevent early PIs after SCI.

In the same line of limitations and with the aim of suggesting new lines of investigation on this topic, it would be interesting to conduct pressure analysis studies comparing both types of cushions with measurements of longer duration. In this regard, it should be noted that the CONFORMat SYSTEM pressure mat records a 30-s interval per measurement. The use of other instruments could enhance the evidence of the dynamic system’s effectiveness in varying maximum pressure areas over a longer time period. Moreover, future prospective studies should be conducted to incorporate clinical outcomes (such as PI incidence, standardized severity grading and infection rates) to complement biomechanical measures and provide a more comprehensive evaluation of cushion performance. Similarly, to test user opinion regarding this type of cushion and to capture its real-world clinical impact, studies could be conducted evaluating the psychosocial impact, usability and user satisfaction with the Nubolo®|Med cushion through various instruments (e.g., Psychosocial Impact of Assistive Devices Scale; PIADS) (34), in cases where participants have used Nubolo®|Med over a more extended trial period.

In conclusion, while previous studies have mainly focused on prototypes or experimental dynamic cushions, this work addresses a critical gap by directly evaluating a commercially available dynamic cushion in comparison with high-performance static cushions. The observed reduction in peak pressure duration at the ischial regions suggests that dynamic redistribution systems may provide a clinically meaningful advantage in pressure injury prevention. These findings emphasize the novelty and practical relevance of testing market-ready devices in real users with spinal cord injury, thereby contributing to evidence-based recommendations for clinical practice.

The results obtained from this study have shown that there are differences in PP an PP/CA values when participants use their usual high-end static air cushion and the Nubolo®|Med cushion. Furthermore, these findings suggest that the dynamic and intelligent Nubolo®|Med cushion has great potential as a tool for the prevention and management of PIs, which, as already mentioned, remain one of the main secondary complications following SCI and represent a significant personal, social, labour, and economic cost for patients and healthcare systems. Importantly, while most previous studies have focused on prototypes or experimental dynamic cushions, this work is, to our knowledge, the first to directly evaluate a commercially available dynamic cushion in comparison with high-performance static cushions. This novelty underscores the clinical and practical relevance of our findings and supports the inclusion of market-ready devices in future research and clinical guidelines. However, as with the evaluation of any preventive or therapeutic tool applied to clinically affected populations, it is important to remember that, although the Nubolo®|Med cushion has shown the ability to reduce PP (which, as discussed, is a key factor in PI prevention), its regular use should not replace the clinical guidelines on PI prevention and management.

## Data Availability

The raw data supporting the conclusions of this article will be made available by the authors, without undue reservation.
