# Supplementary material for: Comparative analysis of sitting pressures in individuals with spinal cord injury: static vs. dynamic air cushions
Source: Front Public Health. 2025 Dec 15;13:1637106. doi: 10.3389/fpubh.2025.1637106 (PMC12745250; doi:10.3389/fpubh.2025.1637106)
Supplement: Supplementary file 1 [file Data_Sheet_1.pdf]

Supplementary material. Comparative results between cushions.

Variable	High-end static cushion	Nubolo®|Med cushion
**Peak Pressure** (PP, mmHg)	38.04 (12.46)	31.81* (9.57)
**Average Pressure** (AP, mmHg)	18.77 (4.24)	18.49 (3.31)
**Contact Area** (CA, mm²)	505.56 (195.68)	519.01 (200.00)
**Peak Pressure normalized by Contact Area** (PP/CA, mmHg/ mm²)	0.080 (0.024)	0.065* (0.016)

*Indicates statistically significant differences when compared with the same variable in the high-end static cushion ($p < 0.001$)
